# Aortic valve insufficiency after Impella device insertion that required aortic valve replacement after Heart Mate III left ventricular assist device implantation: a case report.

**DOI:** 10.1093/jscr/rjab420

**Published:** 2021-10-16

**Authors:** Kohei Ueda, Kenji Yoshitani, Shunsuke Hosotani, Hisanori Hayashi, Satsuki Fukushima, Yoshihiko Ohnishi

**Affiliations:** Department of Anesthesiology, National Cerebral and Cardiovascular Center, Osaka, Japan; Department of Anesthesiology, National Cerebral and Cardiovascular Center, Osaka, Japan; Department of Transfusion, National Cerebral and Cardiovascular Center, Osaka, Japan; Department of Anesthesiology, National Cerebral and Cardiovascular Center, Osaka, Japan; Department of Anesthesiology, National Cerebral and Cardiovascular Center, Osaka, Japan; Department of Cardiovascular Surgery, National Cerebral and Cardiovascular Center, Osaka, Japan; Department of Anesthesiology, National Cerebral and Cardiovascular Center, Osaka, Japan

## Abstract

The Impella (Abiomed, Danvers, MA, USA) is a minimally invasive axial-flow catheter used in severe heart failure. We describe a case in which aortic insufficiency occurred after Impella insertion, required extra surgical intervention twice. A 33-year-old man with familial dilated cardiomyopathy was admitted to our hospital due to acute decompensation of heart failure. Despite intensive medical treatment, his hemodynamic status did not improve. Firstly, Impella was emergently implanted, and HeartMate III (Abbott, Plymouth, MN, USA) implantation was performed 2 weeks after. In the HeartMate III implantation, new aortic insufficiency had revealed and central aortic valve closure was performed concomitantly. However, on postoperative Day1, the coaptation stitch had untied, causing severe aortic insufficiency which led to another emergent operation of aortic valve replacement. We suggest that indications for Impella implantation need to be carefully discussed beforehand, especially with patients who may undergo implantation of left ventricular assist device.

## INTRODUCTION

The Impella device (Abiomed, Danvers, MA, USA) is a minimally invasive axial-flow catheter used in high-risk coronary interventions and for patients in cardiogenic shock. In addition, a recent report has shown that the Impella device can be used to salvage patients in severe heart failure as a bridge to heart transplantation [1]. A recent meta-analysis reported that Impella 5.0 improved short-term outcomes in patients with cardiogenic shock [2]. On the other hand, the Impella device can lead to serious complications such as aortic valve (AV) injury, bleeding, hemolysis and device malfunction. The incidence of AV injury has been reported to be 0.2% [3]. However, once it occurs, it would cause hemodynamic deterioration and require surgical repair.

There is a case report of aortic insufficiency (AI) associated with Impella management that required surgical intervention during left ventricular assist device (LVAD) implantation. In this previous report, AV showed no abnormalities after the concomitant surgery and patients were hemodynamically stabilized with LVAD [4]. However, we report a case of AI caused by Impella insertion and AV closure was performed at the time of LAVD implantation, but the AI recurred the next day and required AV replacement. The case suggests that the insertion of Impella should be carefully considered in patients scheduled for LVAD implantation.

## CASE REPORT

**Figure 1:**
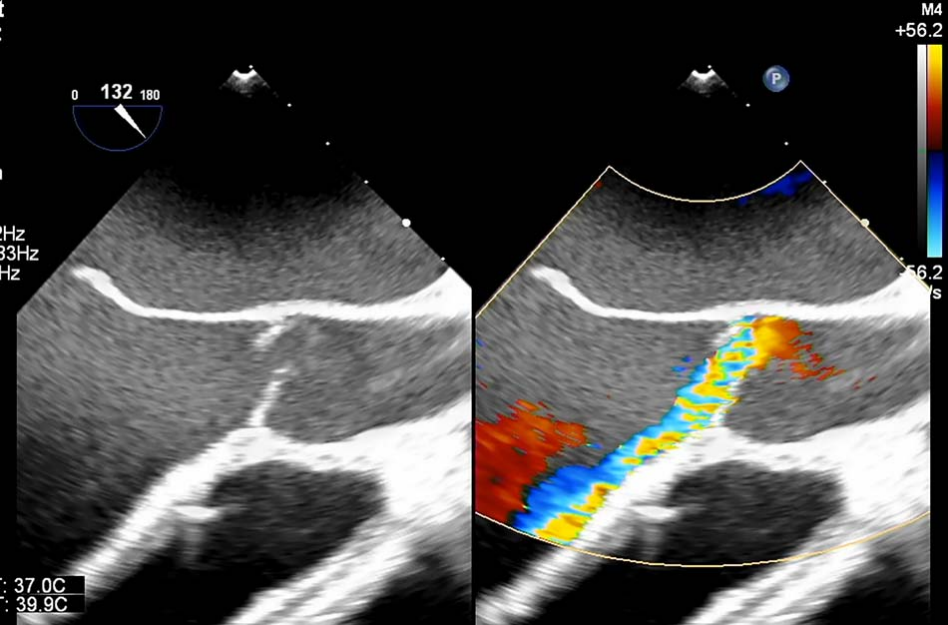
Transesophageal echocardiography showing moderate aortic insufficiency (AI) due to prolapse of noncoronary cusps after Impella removal.

**Figure 2:**
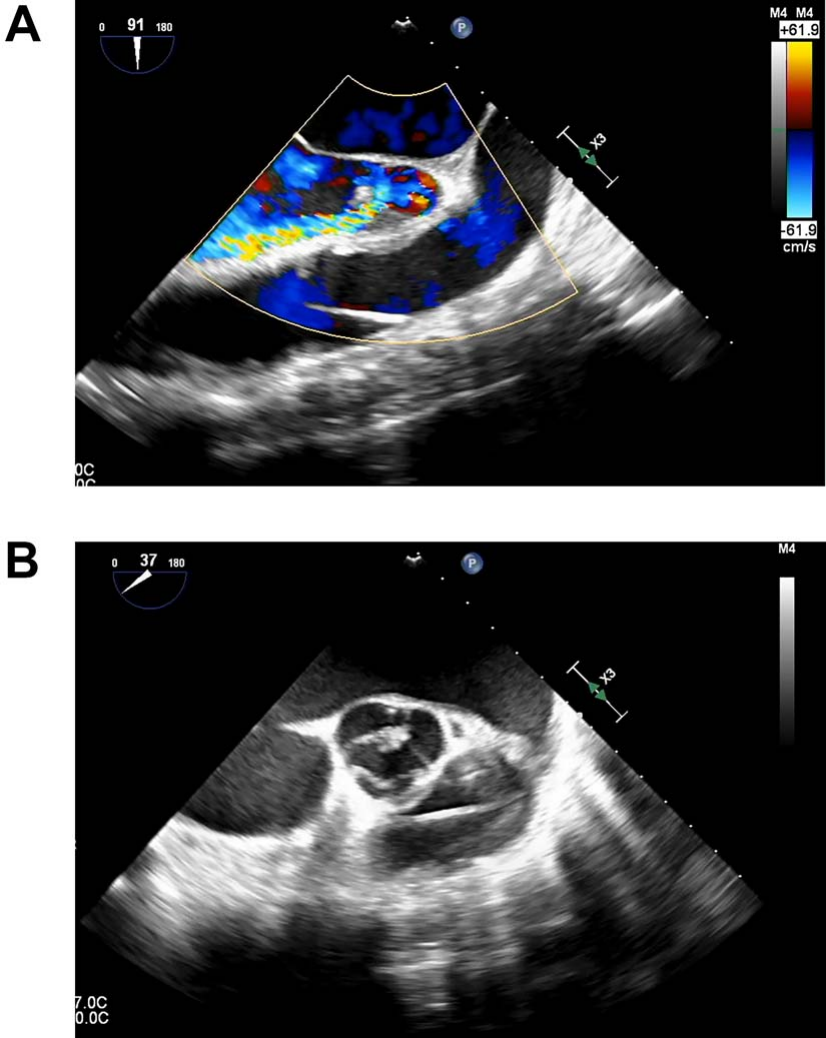
(**A**). Transesophageal echocardiography showing severe AI at 1 day after Heart Mate III implantation. (**B**). Transesophageal echocardiography showing the coaptation stitch in the right and noncoronary cusps had failed at 1 day after Heart Mate III implantation.

A 33-year-old man with familial dilated cardiomyopathy was urgently admitted to our hospital due to acute decompensation of heart failure with brain natriuretic peptide of 1124.5 pg/ml. Transthoracic echocardiography (TEE) showed left ventricular diastolic diameter of 75 mm, left ventricular systolic diameter of 71 mm, left ventricular ejection fraction of 20% with diffuse hypokinesis. Despite intensive medical treatment with dobutamine at 4 mcg/kg/min, his hemodynamic status did not improve: blood pressure (BP) of 91/56 (67) mm Hg, pulmonary artery pressure (PAP) of 56/18(34) mm Hg, pulmonary capillary wedge pressure of 36 mm Hg, central venous pressure (CVP) of 16 mm Hg. An Impella 5.0 catheter was emergently implanted via the right femoral artery as bridging therapy. After the initiation of Impella at the maximum flow rate, TEE showed no AI. The patient’s hemodynamic status stabilized with dopamine at 1.5 mcg/kg/min and dobutamine at 4.2 mcg/kg/min: BP of 79/66(70) mm Hg, PAP of 36/19(25) mm Hg, CVP of 9 mm Hg. Fifteen days after Impella insertion, heart transplantation was approved. HeartMate III (Abbott, Plymouth, MN, USA) implantation was performed. Intraoperative TEE detected mild AI before the Impella device was removed, which worsened to moderate AI after removal due to prolapse of noncoronary cusps (Fig. 1). At that time, BP of 107/48(67) mm Hg, PAP of 68/33(48) mm Hg and CVP of 9 mm Hg. After Park’s stitch procedure (central AV closure) was performed, diastolic arterial BP increased, with BP of 86/60(70) mm Hg, PAP of 41/25(30) mm Hg and CVP of 12 mm Hg. However, on postoperative Day 1, TEE showed the coaptation stitch on the right and noncoronary cusps had failed, causing severe AI (Fig. 2A and B). In addition, BP decreased to 84/42(49) mm Hg. Consequently, emergent AV replacement was performed. Five days after AV replacement, the patient was discharged from the intensive care unit without any complications.

## DISCUSSION

In this case, the Impella device led to AV repair after HeartMate III implantation. Finally, AV replacement was performed after HeartMate III implantation due to malfunction of the repaired AV.

The Impella device has been used frequently for patients with cardiogenic shock as a bridge to durable LVAD implantation. den Uil *et al.* [4] reported that 30% of patients with Impella 5.0 were bridged to durable LVAD with a 30-day survival rate of 63%–100%. However, there is a potential risk of developing AI with Impella implantation. Betsides *et al.* [3] reported that the incidence of AV injury with Impella 5.0 is 0.2%. In another report, the severity of AI worsened in 14.7% of patients, which included 3.3% of patients with new severe AI within 24 hours of Impella device support [5]. Since AI during LVAD support would cause recirculation and decreases LVAD efficiency, surgical treatment would be considered if greater than mild AI had occurred in patients with LVADs [6, 7]. Robertson *et al.* [8] reported that among those who underwent LVAD implantation, 2.3% underwent AV closure, 1.8% underwent AV repair and 1.6% underwent AV replacement as a concomitant procedure. Therefore, it is especially important to assess for the development of AI in patients undergoing LVAD implantation immediately after Impella removal.

Patients with AI after LVAD implantation have higher mortality than those without AI. A recent study found that both short-term and long-term mortality are significantly higher in patients who undergo LVAD implantation with an AV procedure compared with patients who undergo only LVAD implantation (30-day mortality, 10.9% vs. 4.8%; 180-day mortality, 29.1% vs. 15.9%; *P* = 0.001). John *et al.* speculated that need for aortic cross-clamping and cardioplegic arrest contribute to the overall complexity of the procedure and therefore to increased mortality [9]. Therefore, in patients with dilated cardiomyopathy or ischemic cardiomyopathy who are expected to undergo LVAD implantation, the indications for Impella implantation need to be discussed carefully because AI, a serious complication of Impella, would be critical for the patient’s hemodynamics after the LVAD implantation.

Several methods are available for correcting native AI, including AV replacement, complete closure of the ventriculoaortic juncture with a circular patch, partial closure with a single central stitch known as Park’s stitch, and modified Park’s stitch (an additional mattress suture between the central stitch and each commissure) [7]. One report demonstrated that moderate to severe AI recurrence may occur within the first year in up to 18% of patients undergoing LVAD implantation with a concomitant AV procedure (AV repair, 19%; AV closure, 5%; AV replacement, 9%). However, the choice of surgical technique should be decided based on patient characteristics, treatment strategy, anticipated duration of LVAD support and surgical risk [7]. Park’s stitch is relatively simple procedure that can be performed in a short time. On the other hand, it is important to be aware of its short-term durability, compared to complete closure of the ventriculoaortic juncture or AV replacement. AV replacement would be better than Park’s stich in this case. In our case, sutural insufficiency with Park’s stitch occurred on postoperative Day 1, suggesting that AI recurrence may occur very quickly and that careful monitoring for the development of AI during and after Impella removal and LVAD implantation is needed.

## CONCLUSION

In conclusion, Impella implantation would not always be the best choice for patients who may undergo LVAD implantation in the future. The anesthesiologists need to pay attention to the occurrence of new AI after Impella placement. Moreover, if AI occurs and is corrected concomitantly with Impella to LVAD exchange surgery, monitoring for AI recurrence is indispensable.

## CONFLICT OF INTEREST STATEMENT

None declared.
